# Maternal and neonatal outcomes in women with twin pregnancies based on gestational weight gain: An updated systematic review and meta-analysis

**DOI:** 10.12669/pjms.39.4.7529

**Published:** 2023

**Authors:** Xiaoyin Wang, Mei Yan, Zhou Xu, Lin Zhuang

**Affiliations:** 1 Xiaoyin Wang, Department of obstetrical, Hospital of Chengdu University of Traditional Chinese Medicine, Chengdu 610072, Sichuan Province, P.R. China; 2 Mei Yan Department of Gynecology, Sichuan Provincial Maternity and Child Health Care Hospital, Chengdu 610045, Sichuan Province, P.R. China; 3 Zhou Xu, Department of Obstetrical, Si Chuan Jinxin Women and Children Hospital, Chengdu 610011, Sichuan Province, P.R. China; 4 Lin Zhuang, Department of obstetrical, Hospital of Chengdu University of Traditional Chinese Medicine, Chengdu 610072, Sichuan Province, P.R. China

**Keywords:** Twin pregnancy, Gestational weight gain, Meta-analysis, Maternal and neonatal outcomes

## Abstract

**Objective:**

This updated systematic review and meta-analysis aimed to assess maternal and fetal outcomes of pregnancies based on the Institute of Medicine (IOM) guidelines of gestational weight gain (GWG).

**Methods:**

PubMED, SCOPUS, EMBASE and Web of Science were searched up to 30^th^ July 2022. All studies evaluating maternal and/or neonatal outcomes of twin pregnancies based on the IOM guidelines of gestational weight gain were included.

**Results:**

Twenty two studies were included. Mothers with twin pregnancies experiencing inadequate GWG showed higher incidence of gestational diabetes with the risk ratio (RR) 1.22 95% CI (0.95,1.57), p=0.0005, i2= 69% and premature rupture of membrane (PROM) with RR 1.14 95% CI (0.99, 1.30), p=0.07; i2=0% that coincided with higher rates of preterm birth, low birth weight, small for gestational age (SGA) and admission to NICU in neonates. Mothers with excessive GWG had higher risk of developing gestational hypertension with RR 1.59 95% CI (1.22, 2.07), p=0.0006, i2=75% and extremely preterm delivery (<32 weeks).

**Conclusion:**

Within the limitations of this review, GWG was found to be a predictable risk factor for adverse maternal and neonatal outcomes of twin pregnancies.

## INTRODUCTION

Last two to three decades saw an increase in the incidences of twin pregnancies^1^ that are associated with almost 2.5 times higher in-utero mortality and four times higher first-year mortality compared to singleton pregnancies.^2^,^3^ According to the statistics, twins make up 3.2% of all births but account for more than 20% of the burden of preterm birth.^4^ Twin pregnancies put women at higher risk of pre-eclampsia, incidence of gestational diabetes, premature delivery by rupture of membrane, increased risk of caesarean delivery, etc.^5^,^6^ Poor neonatal outcomes that are associated with twin pregnancies include low pre-term birth weight, small for gestational age (SGA) neonates due to restricted fetal growth, perinatal death, and increased need of Neonatal intensive care unit (NICU) admission.^7^ These complications are governed mainly by amount of gestational weight gained by mothers during their pregnancy.^8^

Therefore, the Institute of Medicine (IOM) developed detailed guidelines that define the optimal weight gain for twin pregnancies.^9^ Since the gestational weight gain (GWG) is an easily modifiable factor, controlling it may potentially prevent complications related to adverse outcomes for pregnant women and neonates. Two prior meta-analyses summarized existing analyses of the influence of gestational weight gain on women with twin pregnancies, but the number of included studies was small (11 to 14 studies). Study by Zhong et al. 2021^10^ included 11 reports and concluded that inadequate weight gain in mothers with twin pregnancy led to increased risk of gestational diabetes, reduced risk of hypertension and cesarean delivery among mothers and increased risk of delivery before term, low birthweight (PTLBW), SGA and neonatal intensive unit (NICU) admission. Excessive weight gain was also associated with elevated risk of pre-eclampsia and cesarean section. Study by Lipworth et al. 2022^11^ included 14 manuscripts and concluded that inadequate weight gain leads to fetal growth restriction, and high weight gain leads to gestational diabetes and pre-eclampsia. Current study aims to review new reports that were published over the last two years and to conduct an updated systematic review and meta-analysis to assess maternal and fetal outcomes of twin pregnancies based on the IOM guidelines of GWG.

## METHODS

Preferred Reporting Items for Systematic reviews and Meta-Analyses (PRISMA) 2020 guidelines^12^ were followed. The protocol of the review was registered at PROSPERO, (CRD42022348819).

### Search Strategy:

PubMED, SCOPUS, EMBASE and Web of Science were searched up to 30^th^ July 2022, using relevant keywords: “gestational weight gain”, “twin pregnancy”, “multiple pregnancy”, “maternal outcomes”, “neonatal outcomes”, “pre-natal”, “peri-natal”, “post-natal”. The search strategy used is as follows: ((“gestational weight gain”[All Fields]) AND ((((“twin pregnancy”) OR (“multiple pregnancy”)) OR (double)) OR (twins))) AND ((maternal outcome) OR (neonatal outcome)). Additionally, bibliography of previous systematic reviews and meta-analyses were thoroughly screened for any potentially eligible articles. The citations were deduplicates. Titles and abstracts of the final set of citations were thoroughly screened for eligibility based on relevancy. Full text analysis of the selected studies was then done by the two reviewers based on predefined eligibility criteria. (Supplementary Table-I)

**Supplementary Table-I:** Eligibilty Criteria

Inclusion Criteria	Exclusion Criteria
Studies that evaluated maternal and/or neonatal outcomes of twin pregnancies	Studies that only included singleton pregnancies or pregnancies with higher-order multiples
Studies that included women who were pregnant with twins	Studies that did not report on GWG in twin pregnancies or used a different reference for assessing GWG
Studies that used the Institute of Medicine (IOM) guidelines for gestational weight gain (GWG) as a reference for assessing GWG in twin pregnancies	Studies that focused solely on the effect of maternal BMI or other factors on pregnancy outcomes, without reporting on the relationship between GWG and outcomes in twin pregnancies
Studies that reported on the association between GWG and maternal and/or neonatal outcomes in twin pregnancies	Studies that were not peer-reviewed or were published as conference abstracts, case reports, or letters to the editor
Studies that were published in English	Studies that only included singleton pregnancies or pregnancies with higher-order multiples

### Eligibility Criteria:

All studies evaluating outcomes (both for mothers and neonates) of twin pregnancies based on the IOM guidelines of GWG were included. Studies not reporting relevant outcomes and not following the IOM guideline criteria were excluded.

### Data Collection & Quality assessment:

The data were collected by the two reviewers and the information was fed into the excel spreadsheet (Microsoft Office 365, 2020 version, Microsoft, USA). The study characteristics included demographic data such as study design, duration, and setting, sample size, assessed outcomes, Body mass index (BMI) stratification, GWG and number of participants per group. The maternal outcomes i.e., incidence of gestational diabetes, gestation hypertension, caesarean delivery, post-partum hemorrhage, PROM and neonatal outcomes like pre-term birth at 37 and 32 weeks, SGA, low birth weight (less than 2500gm), and admission to NICU, whichever available, were collected. Quality of the included studies was assessed by the New-castle Ottawa Scale (NOS).^13^

### Data Analysis:

The maternal and neonatal outcomes data were dichotomous and combined to generate pooled risk ratio (RR) with 95% confidence interval (CI) using RevMan (Review Manager software). The heterogeneity among the included studies were calculated using I2 statistics. A sensitivity analysis was performed to check influence of each study on the outcome.

## RESULTS

**Supplementary Fig.1:**
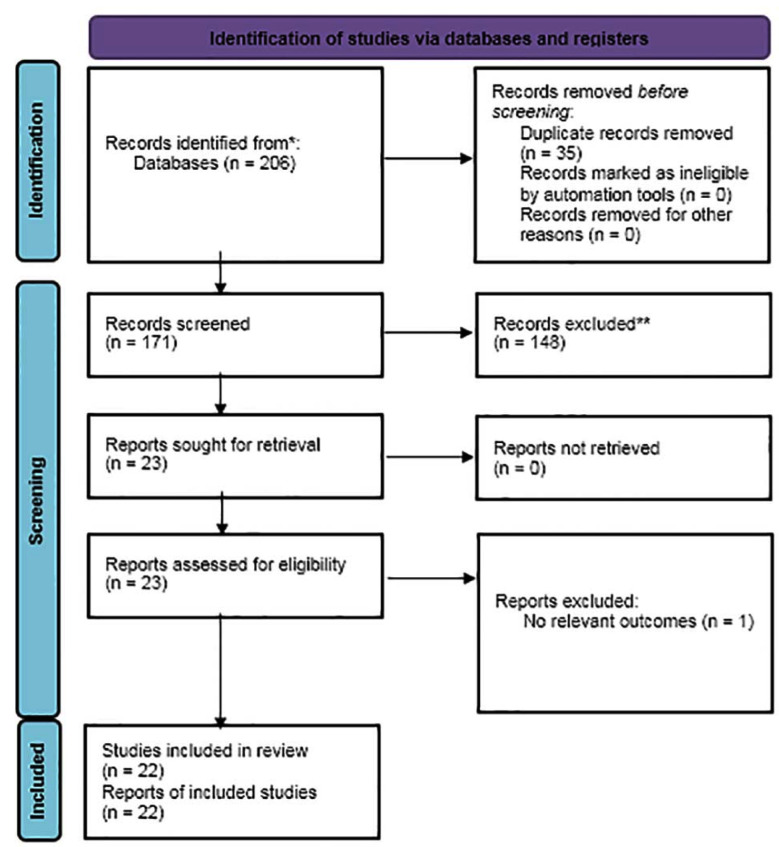
PRISMA flow chart depicting the study selection process.

A total of 22 studies^14^–^35^ were included in this review. The comprehensive search of digital databases and hand search identified a total of 206 citations. Duplicates were removed and title and abstract of the remaining 171 citations were reviewed. Finally, full text assessment was done on 23 records, of which, twenty-two studies were finally included in the current review.(Supplementary Fig.1)

All the included studies were retrospective in design. Data on total of 74222 mothers with twin pregnancy were reported. Of them, 43,765 mothers had inadequate GWG below IOM range of weight gain and 30,457 mothers had excessive GWG above the IOM range. In few studies participants were also stratified according to BMI. As summarized in Table-I, a total of 4567 mothers were under-weight, 16789 were of normal weight, 15678 were overweight and 6546 were obese. The mean age of the included mothers was 25.6±7.7 years. All included studies were of good quality.(Supplementary Table-II)

**Table-I:** Demographic characteristics of included studies

SL. No.	Author	Year	Study design	Study Duration	No. of Women	GWG		NOS score

						Below IOM	Above IOM	
1	Lin et al.	2022	Retrospective study	2014 to 2018	931	309	269	8
2	Maeda et al.	2022	Retrospective study	2007 to 2015	NW-17973, UW-4394	UW-11.79(4.64), NW-11.49(4.83)		
3	Lipworth et al.	2021	Retrospective cohort study	2000 to 2014	1274 (UW-43, NW-777, OW-278, Obese-176)	UW-14(33), NW-238(31), OW-71(26), Obese-58(33)	UW-4(9), NW-121(16), OW-61(22), Obese-30(17)	8
4	Liu et al.	2021	Retrospective cohort study	2005 to 2017	609	223	NR	7
5	Choi et al.	2020	Retrospective study	2005 to 2019	1738	881	163	8
6	Shimura et al	2020	Retrospective study	2006 to 2018	265	226	NR	8
7	Bodnar et al.	2019	population-based cohort study	2003 to 2013	27.723	17+/-8.5		
8	Lin et al.	2019	Retrospective cohort study	2015 to 2018	645	97	281	8
9	Pecheux et al.	2019	Retrospective cohort study	1997 to 2013	878	468	64	8
10	Algeri et al.	2018	Retrospective cohort study	2010 to 2013	175	91	11	9
11	Wang et al.	2018	Retrospective cohort study	2015 to 2016	350	145	35	8
12	Kosinska-Kaczynska et al.	2017	Prospective cohort study	2007 to 2016	295	77	25	8
13	Lutsiv et al.	2017	Retrospective cohort study	2003 to 2014	741	201	220	7
14	Ozcan et al.	2016	Retrospective cohort study	2004 to 2014	5897	NR	NR	7
15	Pettit et al.	2015	Retrospective cohort study	2001 to 2014	489	NR	203	8
16	Lal et al.	2015	Retrospective cohort study	2002 to 2008	2654	1040	517	7
17	Shamshiraz et al.	2014	Retrospective cohort study	1991 to 2011	570	NR	NR	8
18	Pettit et al.	2014	Retrospective cohort study	2001 to 2013	489	93	NR	8
19	Gavard et al.	2014	Population-based historical cohort study	1998 to 2005	831	256	252	8
20	Gonzalez-Quintero et al.	2012	Retrospective study		5129	n=1366; 23.4+/- 8.4 pounds	n=1646; 47.7 +/-13.3 pounds	8
21	Fox et al.	2011	Retrospective cohort study	2005 to 2010	170	55	39	9
22	Fox et al.	2010	Cohort study	2005 to 2009	297	105	NR	8

**Supplementary Table II:** Quality of included studies.

Study	Year	Selection			Comparability			Outcome		

		Representativeness of the exposed cohort	Selection of the nonexposed cohort	Ascertainment of exposure	Demonstration that outcome of interest	Basis of the design or analysis	Assessment of outcome	follow-up long enough for outcomes	Adequate follow up	Total
Lin et al.	2022	1	1	1	1	1	1	1	1	8
Maeda et al.	2022	1	1	1	1	1	1	1	1	8
Lipworth et al.	2021	1	1	1	1	1	1	1	1	8
Liu et al.	2021	1	1	0	1	1	1	1	1	7
Choi et al.	2020	1	1	1	1	1	1	1	1	8
Shimura et al	2020	1	1	1	1	1	1	1	1	8
Bodnar et al.	2019	1	1	1	1	1	1	1	1	8
Lin et al.	2019	1	1	1	1	1	1	1	1	8
Pecheux et al.	2019	1	1	1	1	1	1	1	1	8
Algeri et al.	2018	1	1	1	1	2	1	1	1	9
Wang et al.	2018	1	1	1	1	1	1	1	1	8
Lutsiv et al.	2017	1	1	0	1	1	1	1	1	7
Ozcan et al.	2016	1	1	0	1	1	1	1	1	7
Pettit et al.	2015	1	1	1	1	1	1	1	1	8
Lal et al.	2015	1	1	0	1	1	1	1	1	7
Shamshiraz et al.	2014	1	1	1	1	1	1	1	1	8
Pettit et al.	2014	1	1	1	1	1	1	1	1	8
Gavard et al.	2014	1	1	1	1	1	1	1	1	8
Gonzalez-Quintero et al.	2012	1	1	1	1	1	1	1	1	8
Fox et al.	2011	1	1	1	1	2	1	1	1	9
Fox et al.	2010	1	1	1	1	1	1	1	1	8

### Meta-Analysis:

### Inadequate GWG versus Adequate GWG:

**Fig.1:**
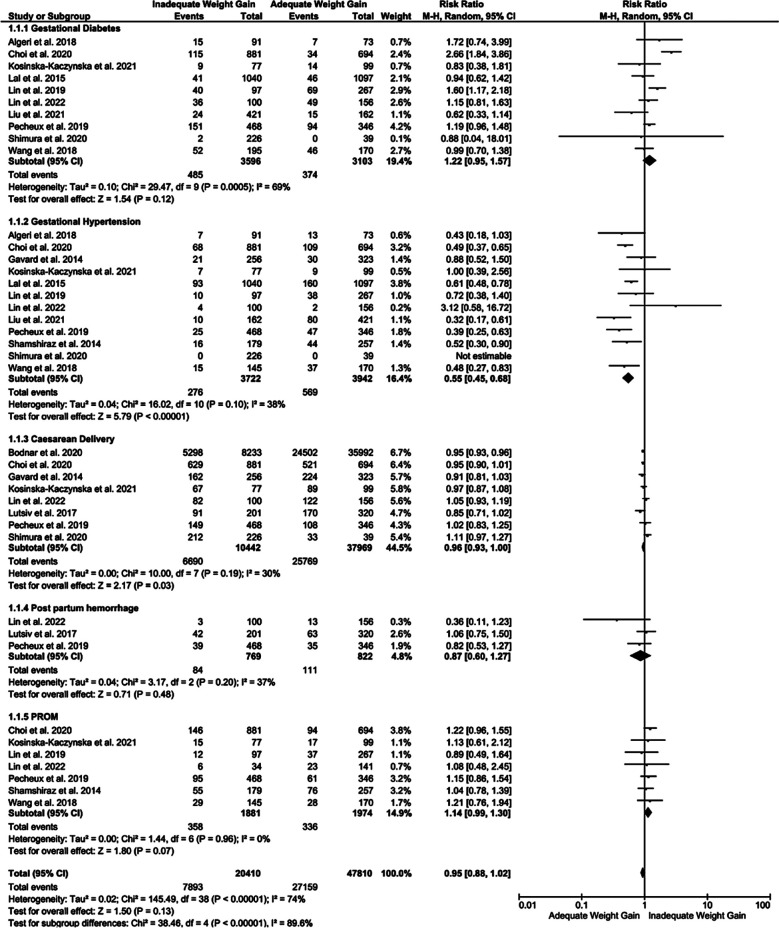
Forest plot showing comparison of maternal outcomes among the mothers with twin pregnancy showing inadequate or adequate GWG.

Inadequate GWG correlated with higher incidence of gestational diabetes in women with twin pregnancies (RR 1.22, 95% CI [0.95,1.57], p=0.0005) with moderate heterogeneity (i2= 69%). However, the risk of developing gestational hypertension was low among the mothers with twin pregnancy with inadequate GWG, with RR of 0.55 95% CI [0.45,0.68], p<0.0001, i2=38%. Inadequate GWG also correlated with lower risk of caesarian delivery compared to mothers with adequate GWG (RR of 0.96). There was no difference in the effect estimate for post-partum hemorrhage between mothers with inadequate GWG compared to adequate GWG with RR 0.87 95% CI [0.60,1.27], p=0.48. As shown in Fig.1, the incidence of PROM was also higher in women with twin pregnancies with inadequate GWG (RR of 1.14 95% CI [0.99, 1.30], p=0.07; i2=0%).

**Fig.2:**
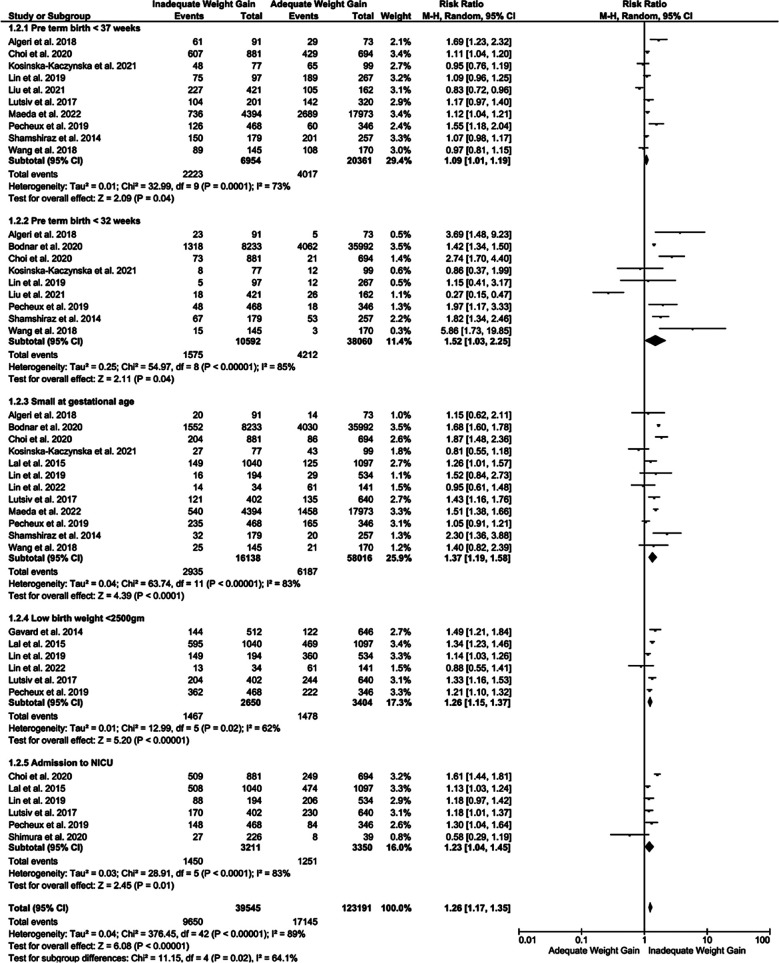
Forest plot showing comparison of neonatal outcomes among the mothers with twin pregnancy showing inadequate or adequate GWG.

In terms of the neonatal outcomes, women with inadequate GWG were at higher risk of preterm (<37 weeks) and very preterm (<32 weeks) delivery with RR 1.09 (p=0.04) and RR 1.52 (p=0.04), respectively. Similarly, risk of SGA, low birth weight neonates (<2500 gm) and risk of neonatal admission to NICU immediately after birth was higher [RR 1.37 (p<0.001), 1.26 (p<0.001) and 1.23 (p<0.02)], respectively, in cases of inadequate GWG compared adequate GWG (Fig.2).

### Excessive GWG versus Adequate GWG:

**Fig.3:**
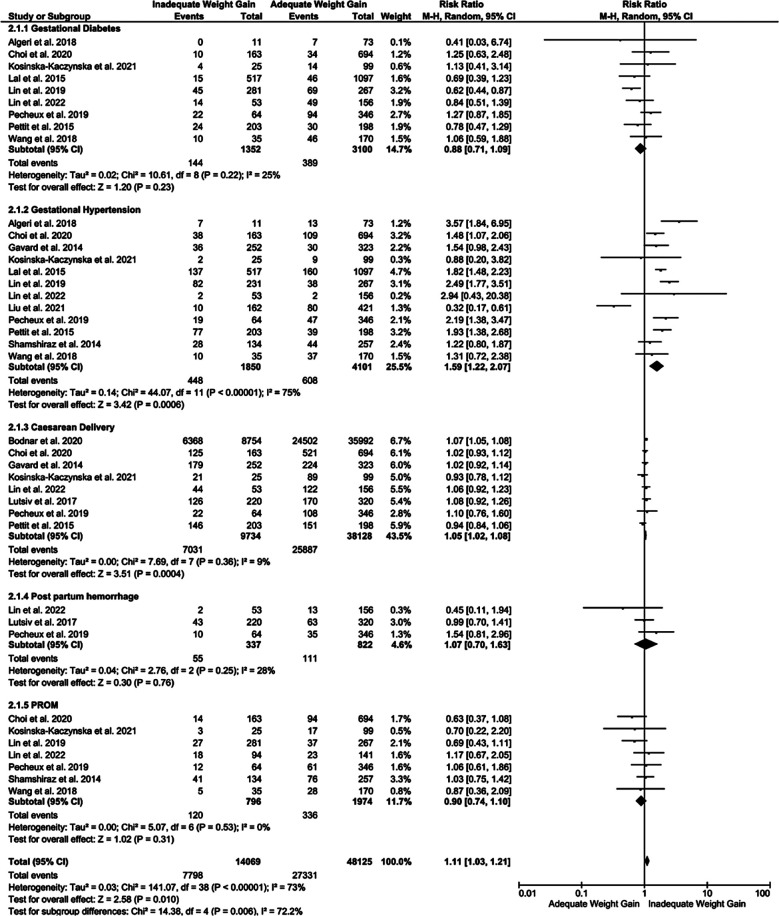
Forest plot showing comparison of maternal outcomes among the mothers with twin pregnancy showing excessive or adequate GWG.

The risk of developing gestational hypertension was higher among mothers with twin pregnancy with excessive GWG [RR of 1.59 95% CI [1.22, 2.07], p=0.0006, i2=75%], compared to mothers with adequate GWG. However, no major difference was observed in the incidence of gestational diabetes, caesarean delivery, post-partum hemorrhage and PROM (Fig.3).

**Fig.4:**
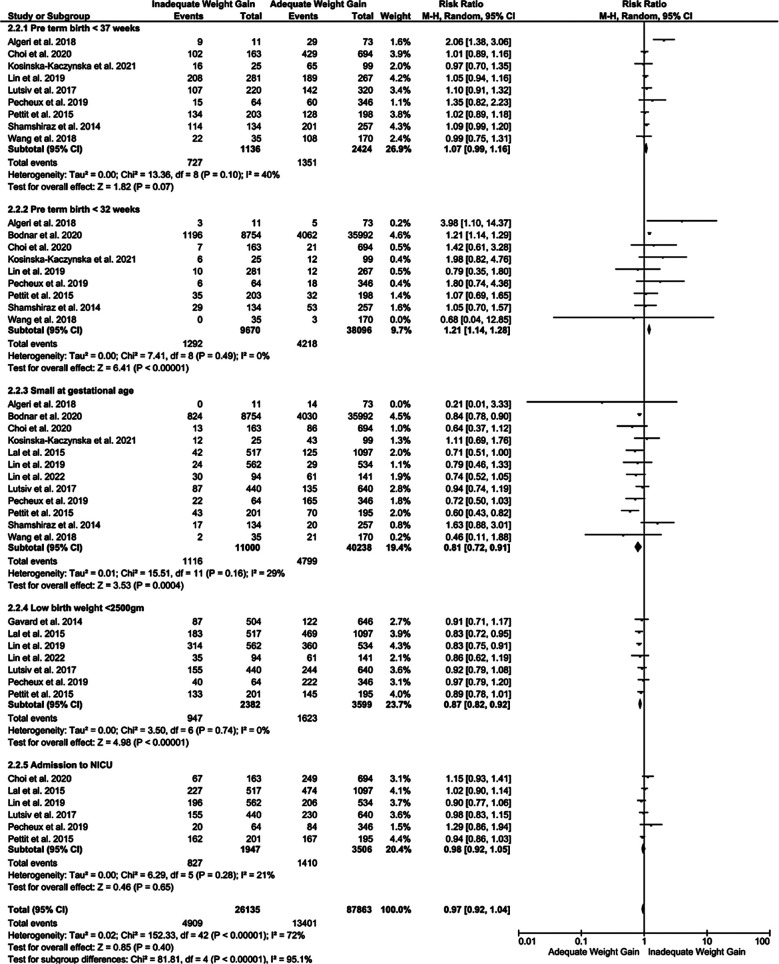
Forest plot showing comparison of neonatal outcomes among the mothers with twin pregnancy showing excessive or adequate GWG.

Excessive GWG in mother with twin pregnancy correlated with significantly higher incidence of very preterm (<32 weeks) birth [RR of 1.21 95% CI [1.14, 1.28], p<0.0001, i2=0%], compared to women with adequate GWH. The rate of preterm delivery (< 37 weeks) and admission to NICU was similar between the groups. Additionally, the risk of delivering SGA and low birth weight neonates was also low (RR 0.81 and 0.87 respectively) in mothers with twin pregnancy and excessive GWG compared to adequate GWG (Fig.4). Sensitivity analysis identified the outliers which could possibly change the effect estimate based on their weight. (Refer Supplementary Table-III).

**Supplementary Table-III:** Sensitivity analysis of the effect estimate of each outcome by toggling through each included study.

	Outcomes	Studies included		Change in significance of RR
1	*Comparison of outcomes between Inadequate GWG versus Adequate GWG in twin mothers*			
1.1	Maternal Outcomes			
1.1.1	Gestational Diabetes	9	Removing other studies	No Major Change
			Removing Liu et al. 2021	1.30 [1.01, 1.66], *p*=0.04
1.1.2	Gestational Hypertension	12	Removing each study	No Major Change
1.1.3	Caesarean Delivery	8	Removing Other studies	No Major Change
			Removing Bodnar et al. 2020	0.97 [0.92, 1.03], p=0.37
			Removing Choi et al. 2020	0.97 [0.92, 1.02], p=0.23
			Removing Shimura et al. 2020	0.95 [0.93, 0.96], p<0.0001
1.1.4	Post-partum haemorrhage	3	Removing each study	No Major Change
1.1.5	Premature rupture of membrane	6	Removing other studies	No Major Change
			Removing Lin et al. 2019	1.15 [1.00, 1.33], p=0.05
1.2	Neonatal Outcomes			
1.2.1	Pre-term birth <37 weeks	10	Removing each study	No Major Change
1.2.2	Pre-term birth <32 weeks	8	Removing other studies	No Major Change
			Removing Liu et al. 2021	1.87 [1.40, 2.50], p<0.0001
1.2.3	Small at Gestational Age	12	Removing each study	No Major Change
1.2.4	Low birth weight <2500gm	6	Removing each study	No Major Change
1.2.5	Admission to NICU	6	Removing each study	No Major Change
2	*Comparison of outcomes between Excessive GWG versus Adequate GWG in twin mothers*			
2.1	Maternal Outcomes			
2.1.1	Gestational Diabetes	9	Removing other studies	No Major Change
			Removing Pecheux et al. 2019	0.78 [0.64, 0.95], p<0.01
2.1.2	Gestational Hypertension	12	Removing each study	No Major Change
2.1.3	Caesarean Delivery	8	Removing Other studies	No Major Change
			Removing Bodnar et al. 2020	1.05 [1.02, 1.08], p=0.0004
2.1.4	Post-partum haemorrhage	3	Removing each study	No Major Change
2.1.5	Premature rupture of membrane	7	Removing each study	No Major Change
2.2	Neonatal Outcomes			
2.2.1	Pre-term birth <37 weeks	9	Removing each study	No Major Change
2.2.2	Pre-term birth <32 weeks	9	Removing each study	No Major Change
2.2.3	Small at Gestational Age	12	Removing each study	No Major Change
2.2.4	Low birth weight <2500gm	7	Removing each study	No Major Change
2.2.5	Admission to NICU	6	Removing each study	No Major Change

## DISCUSSION

Our study aimed to evaluate the maternal and neonatal outcomes of twin pregnancies in women with inadequate or excessive GWG compared to the normal range of GWG as delineated by the IOM guidelines for twin pregnancy. The results of this systematic review were derived from the pooled estimate of twenty-one studies reporting maternal and neonatal outcomes, with two or more studies available for each outcome. Our result showed increased rates of gestational diabetes and PROM in mothers with twin pregnancies and inadequate GWG. Additionally, neonates from mothers with twin pregnancies and inadequate GWG had higher risk of being delivered preterm (at less than 37 and 32 weeks), being SGA, and low birth weight. Inadequate GMG coincided with higher rate of neonatal admission to NICU at birth. Mothers with twin pregnancies and excessive GWG has higher risk of developing gestational hypertension. Additionally, excessive GWG was associated with slightly higher (but not statistically significant) risks of cesarean delivery and post-partum hemorrhage.

Inadequate GWG is typically associated with nutritional deficiencies, insufficient plasma volume expansion, and metabolic state that could prevent the weight gain.^36^ These factors may also increase the risk of infection or inflammation. The state of nutritional deficiency increases women’s susceptibility to a wide range of infection, which may directly or indirectly affect the maternal and fetal outcomes.^37^ The state of long-term infection in mothers with inadequate GWG may lead to the risk of preterm delivery due to early oxytocin release in the presence of pro-inflammatory cytokines. In addition, this state of surge in inflammatory cytokines is also responsible for the release of certain proteins and interleukins which inhibit the insulin signaling pathways, interfering with the insulin release and leading to potentially elevated blood sugar levels and gestational diabetes.^38^

Excessive GWG also possesses a risk for pro-inflammatory state in mothers due to the release of pro-inflammatory cytokines from adipocytes. The increase in the acute phase reactants and pro-inflammatory cytokines could induce PROMs and vasoconstriction, leading to relative rise in the blood pressure. Therefore, excessive GWG in mothers with high BMI may result in a state of preeclampsia, where the blood pressure rises abnormally, leading to various complications.^39^

The results of our updated systematic review and meta-analysis confirm findings of previous systematic reviews by Zhong et al.^10^particularly in singleton pregnancies, has been well-linked with maternal and infant outcomes. The aim of the current meta-analysis was to evaluate the effects of gestational weight gain on maternal and fetal outcomes in women with twin pregnancies., Methods: A systematic search was conducted using the PubMed, Scopus, and Google Scholar databases. Studies, either retrospective or prospective in design, evaluating the effects of gestational weight gain (defined using Institute of Medicine (IOM and Lipworth et al.^11^ Study by Zhong et al.^10^particularly in singleton pregnancies, has been well-linked with maternal and infant outcomes. The aim of the current meta-analysis was to evaluate the effects of gestational weight gain on maternal and fetal outcomes in women with twin pregnancies., Methods: A systematic search was conducted using the PubMed, Scopus, and Google Scholar databases. Studies, either retrospective or prospective in design, evaluating the effects of gestational weight gain (defined using Institute of Medicine (IOM included eleven papers and concluded that inadequate weight gain in mothers with twin pregnancy led to increased risk of gestational diabetes, reduced risk of hypertension and cesarean delivery among mothers and increased risk of PTLBW^38^, SGA and NICU admission in neonates. Moreover, excessive weight gain was associated with increased risk of preeclampsia and cesarean delivery. Lipworth et al.^11^ included fourteen studies and concluded that inadequate weight gain led to fetal growth restriction and high weight gain lead to gestational diabetes and preeclampsia.

### Limitations:

All included studies were retrospective observational with low number of participants. Moreover, most of the included studies did not take into account variables such as BMI, age, chorionicity of twins, assisted reproductive technologies, accessibility to medical facilities etc that can potentially be cofounding. The BMI of mothers prior to gestation is an important risk factor in the mothers with twin pregnancy.^39^ The attempt of BMI stratification was carried out to provide a more comprehensive result based on prepartum BMI. Few studies had a clear distinction of underweight, normal weight, overweight and obese mothers. However, the results provided by these studies were not stratified accordingly.

Therefore, we did not attempt a sub-group analysis based on BMI. The number of included studies was not enough to justify the use of subgroup analysis in rest of the outcomes with most of the outcomes included in one study. Other potential confounding factors that could have influenced the results of the included studies include maternal age, parity, ethnicity, and socioeconomic status, as well as the number and chorionicity of the twin fetuses. These factors have been shown to be associated with GWG and pregnancy outcomes in general, and may also be relevant in the context of twin pregnancies.

The age of the mothers with twin pregnancy and chorionicity of twins are also among risk factors which have to be considered while evaluating these maternal and neonatal outcomes. Additionally, the included studies varied in terms of their study design, sample size, and methodological quality, which could have also affected the results. For example, some studies may have had a higher risk of bias due to incomplete outcome reporting or confounding factors that were not adequately controlled for. Further prospective and other observational studies are required to strengthen the evidence by considering the limitations mentioned above.

## CONCLUSION

GWG was found to be a predictable risk factor for adverse maternal and neonatal outcomes in mothers with twin pregnancies. Inadequate GWG correlated with increased rates of gestational diabetes and PROM in women with twin pregnancies, as well as preterm birth, low birth weight, SGA and admission to NICU in neonates. Women with excessive GWG had higher risk of developing gestational hypertension and very preterm (at <32 weeks) delivery. As GWG is a potentially modifiable factor, mothers with twin pregnancies should be counselled on the importance of optimal GWG to prevent potential adverse maternal and fetal/neonatal outcomes. Healthcare providers should educate mothers on the IOM guidelines for GWG and provide individualized recommendations based on their pre-pregnancy BMI and other relevant factors. Regular monitoring of weight gain during prenatal visits can also help identify mothers who may be at risk of inadequate or excessive GWG and provide appropriate interventions.

### Authors’ contributions:

**XW** conceived and designed the study.

**MY, ZX and LZ** collected the data and performed the analysis.

**XW** was involved in the writing of the manuscript and is responsible for the integrity of the study.

All authors have read and approved the final manuscript.
